# Enhanced Supercapacitive Performance of Higher-Ordered 3D-Hierarchical Structures of Hydrothermally Obtained ZnCo_2_O_4_ for Energy Storage Devices

**DOI:** 10.3390/nano10061206

**Published:** 2020-06-19

**Authors:** Gutturu Rajasekhara Reddy, Nadavala Siva Kumar, Borelli Deva Prasad Raju, Gnanendra Shanmugam, Ebrahim H. Al-Ghurabi, Mohammad Asif

**Affiliations:** 1 Department of Instrumentation, Sri Venkateswara University, Tirupati 517502, India; guttururajasekharareddy@gmail.com; 2 Department of Chemical Engineering, King Saud University, P.O. Box 800, Riyadh 11421, Saudi Arabia; alghurabi83@windowslive.com (E.H.A.-G.); masif@ksu.edu.sa (M.A.); 3 Department of Physics, Sri Venkateswara University, Tirupati 517 502, India; 4 Department of Biotechnology, Yeungnam University, Gyeongsan, Gyeongbuk 38541, Korea

**Keywords:** 2D-nanoflakes, 3D-hierarchical peony-like structures, ZnCo_2_O_4_, supercapacitors

## Abstract

The demand for eco-friendly renewable energy resources as energy storage and management devices is increased due to their high-power density and fast charge/discharge capacity. Recently, supercapacitors have fascinated due to their fast charge–discharge capability and high-power density along with safety. Herein, the authors present the synthesis of 3D-hierarchical peony-like ZnCo_2_O_4_ structures with 2D-nanoflakes by a hydrothermal method using polyvinylpyrrolidone. The reaction time was modified to obtain two samples (ZCO-6h and ZCO-12h) and the rest of the synthesis conditions were the same. The synthesized structures were systematically studied through various techniques: their crystalline characteristics were studied through XRD analysis, their morphologies were inspected through SEM and TEM, and the elemental distribution and oxidation states were studied by X-ray photoelectron spectroscopy (XPS). ZCO-12h sample has a larger surface area (55.40 m^2^·g^−1^) and pore size (24.69 nm) than ZCO-6h, enabling high-speed transport of ions and electrons. The ZCO-12h electrode showed a high-specific capacitance of 421.05 F·g^−1^ (31.52 C·g^−1^) at 1 A·g^−1^ and excellent cycle performance as measured by electrochemical analysis. Moreover, the morphologic characteristics of the prepared hierarchical materials contributed significantly to the improvement of specific capacitance. The excellent capacitive outcomes recommend the 3D-ZnCo_2_O_4_ hierarchical peony-like structures composed of 2D-nanoflakes as promising materials for supercapacitors with high-performance.

## 1. Introduction

Over the past few decades, energy management, transportation and combination have become crucial for the rapidly growing market owing to the depletion of fossil fuel and the development of eco-friendly energy resources [1,2,3,4]. In this regard, clean, inexpensive, and consistent energy technologies such as batteries, and supercapacitors (SCs) have come into the limelight in our modern day-to-day life [5,6,7]. Recently, SCs are considered promising energy resources owing to excellent reliability, high power density, better setup safety, fast charge/discharge capability and large energy density [8,9,10]. The SCs can be divided into electrochemical double-layer capacitors (EDLC) or pseudocapacitors [11]. In EDLCs, the electrode is composed of CNT, activated carbon, graphene and other carbon-oriented materials, which can supply energy through ion adsorption on a material surface [12,13]. Moreover, the carbon materials have a large specific surface area, good chemical stability and good electronic conduction. Differently, the pseudocapacitors depend on the fast-reversible faradic redox reactions at the surface material of the electrode, which provides a high theoretical capacitance based on the multiple oxidation states and morphology [14,15]. Thus, numerous studies have focused on electrode materials such as conducting polymers, binary and ternary transition metal oxides [16,17,18]. Based on these ternary metal oxides such as NiCo_2_O_4_ [19], CuCo_2_O_4_ [20], ZnCo_2_O_4_ [21], NiMoO_4_ [22], Zn_2_SO_4_ [23] and NiFe_2_O_4_ [24] show fascinating electrochemical performances due to the synergetic effects of the metal ions. ZnCo_2_O_4_ (ZCO) has been promoted as an electrode material for energy storage devices due to its high theoretical capacitance, electrical conductance, enhanced cycle stability, abundant resources and low price [25,26,27]. Recently, ZCO has been studied for Li-ion batteries and photocatalytic CO_2_ reduction [28,29]. ZCO materials have been prepared with various morphologies such as nanosheets, nanorods, nanotubes, nanowires and nanoparticles [30,31]. Furthermore, microstructures prepared for Li-ion batteries consist of nanosheets, mesoporous structures, yolk–shell structures and microspheres [32]. In recent times, 3D-well-organized microstructures comprising nanoarchitectures have emerged as encouraging constituents for energy storage applications owing to their robust mechanical stability and a short diffusion path of ions and electrons [33,34].

Herein, polyvinylpyrrolidone (PVP)-supported ZCO 3D-hierarchical peony-like structures were fabricated by a hydrothermal route and used for pseudocapacitor with high-performance. The electrode had a 3D-structure consisting of 2D-nanoflakes with high specific capacitance and showed good cycle stability. The prepared materials exhibited notable electrochemical performances because the ZCO has hierarchical structures consisting of 2D-nanoflakes.

## 2. Materials and Methods

### Fabrication of 3D-Peony-Like ZCO

All chemical reagents were procured from Sigma-Aldrich, St. Louis, MO, USA. First, 0.1 g of PVP (K-30) was added with 240 mL of deionized (DI) water and apply constant stirring to get a homogeneous solution. Then, 0.7 g (10 mmol) of zinc nitrate hexahydrate, 1.4 g (20 mmol) of cobalt nitrate hexahydrate and 0.7 g (48 mmol) of urea were mixed to the PVP solution with stirring. After stirring for a further 30 min, the mixed solution was shifted to a hydrothermal reactor (Zhengzhou Keda Machinery, Zhengzhou, Henan, China) and maintained at 180 °C for 6 h in the furnace. After cooling the reactor, the precipitate was collected, cleaned with ethanol and DI water and lastly dried at 80 °C for 10 h. Afterward, the products were annealed at 400 °C under an air atmosphere for 4 h with a ramping rate of 5 °C /min to acquire crystallized flower-like ZCO (denoted as ZCO-6h). Flake-like ZCO (denoted as ZCO-12h) was produced under similar synthesis conditions, except for the reaction time (180 °C/12 h). The material characterization and electrochemical measurements are described in the Supplementary Materials.

## 3. Results and Discussion

### 3.1. XRD Analysis

**Figure 1:**
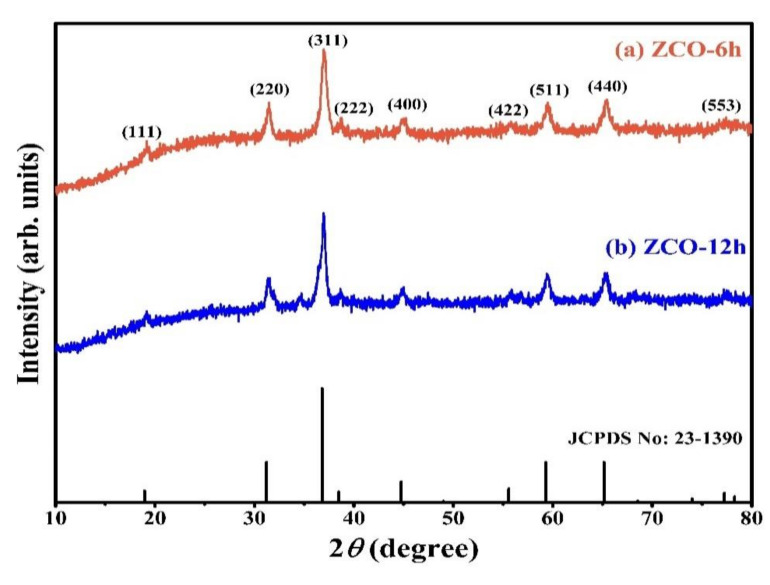
XRD pattern of the as-prepared ZCO-6h and ZCO-12h.

XRD profiles (PANalytical X’Pert PRO, Malvern, UK) of the as-prepared ZCO-6h and ZCO-12h samples match well with the standard XRD profile of ZCO (Figure 1) (JCPDS No.: 23–1390) [21,26]. The Bragg’s diffraction peaks appeared at 18.80°, 31.19°, 36.78°, 44.5°, 55.58°, 59.27° and 65.09° corresponding to the reflections of (111), (220), (311), (222), (400), (422), (511) and (440), respectively. No additional impurity peaks were detected in the XRD profiles, which indicates that the crystal structure of the prepared ZCO is uncontaminated. Furthermore, the average crystallite size of ZCO-6h and ZCO-12h was calculated as 15 and 14 nm, respectively, using Scherrer’s equation [35].

### 3.2. Morphologic Analysis

**Figure 2:**
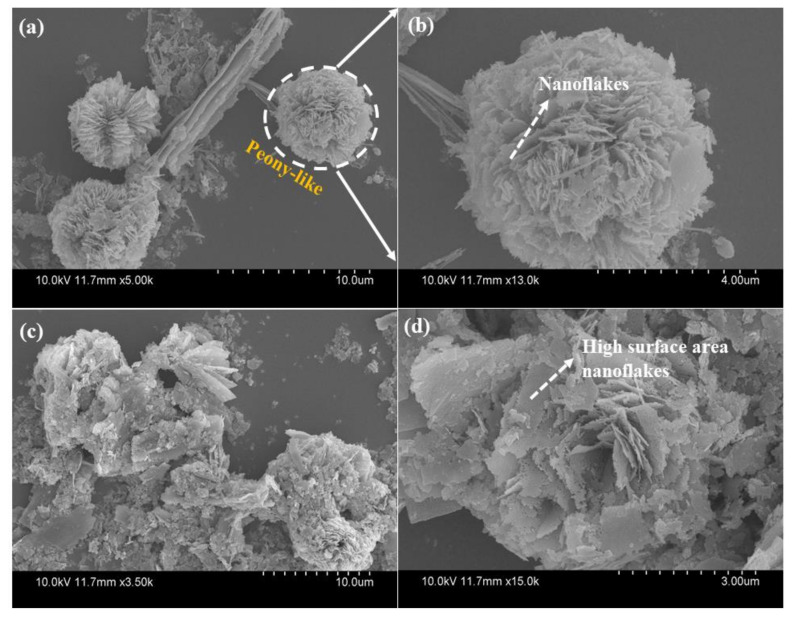
Field emission scanning electron microscopy (FE-SEM) images of ZCO-6h (**a**,**b**) and ZCO-12h (**c**,**d**) samples at different magnifications.

The size, morphology and assembly of the ZCO structure were examined by FE-SEM (Hitachi, S-4800, Tokyo, Japan) coupled with EDS and TEM studies. The SEM images (Figure 2a,b) of the as-prepared ZCO-6h shows 3D-hierarchical peony-like architectures of different sizes, with the petals made up of densely interconnected nanosized flakes with a diameter of 20–30 nm. The high-magnification SEM image displays that the surface of the ZCO-6h sample is porous and rough, and assembled with numerous nanoflakes. These nanoflakes are composed of small nanoparticles. Furthermore, these nanoflakes are self-assembled to form peony-like architectures, which leads to improves the mechanical strength and generate crevices for the quick response of electrolyte ions within the redox phase, resulting for improving electrochemical properties [36,37]. To further examine the morphology of ZCO-12h, the sample was synthesized at 180 °C/12 h, as displayed in Figure 2c,d. In the case of 12 h reaction time, the flower-like structure undergoes fragmentation, leading to the formation of individual agglomerated nanoflakes.

**Figure 3:**
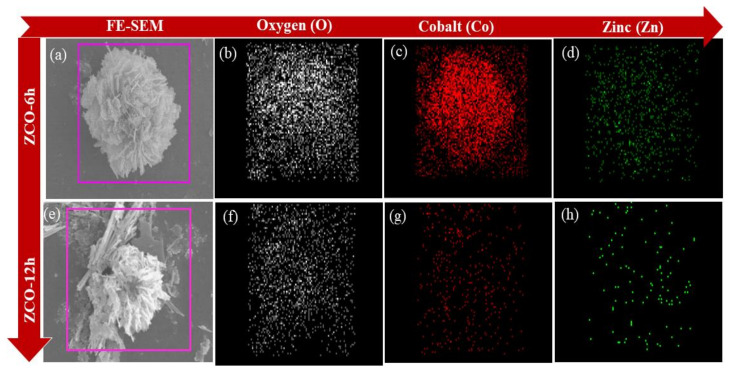
FE-SEM images for ZCO-6h (**a**) and ZCO-12h (**e**) and their respective elemental mapping images of (**b**,**f**) oxygen (O), (**c**,**g**) Cobalt (Co) and (**d**,**h**) Zinc (Zn).

Furthermore, PVP exhibits a substantial part in the formation of the peony-like morphology of ZCO structures. Due to the coordination effect of PVP, the morphologies of metal oxides were controlled [3]. Because PVP molecules contain both hydrophilic and hydrophobic functionality, they can prevent particles from agglomerating with each other, making them an excellent surface stabilizer, that can change the dynamic growth of specific crystal planes and act as a morphology control agent. Furthermore, the elemental percentage of the ZCO-6h and ZCO-12h were qualitatively examined by FE-SEM coupled with EDS, as exposed in Figure 3. For two samples, it is obvious that the uniform distribution of each component mapped images, such as O (white), Co (red) and Zn (green). The mapping images of the ZCO-6h and ZCO-12h samples are depicted in Figure 3a–d and 3e–h, respectively.

**Figure 4:**
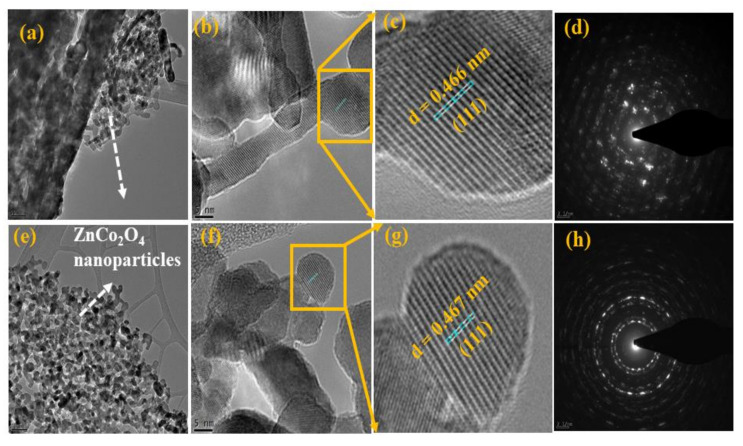
TEM and HR-TEM images and the corresponding SAED patterns for (**a**–**d**) ZCO-6h and (**e**–**h**) ZCO-12h.

Moreover, the morphology and peony-like structures of the prepared ZCO were determined by HR-TEM (Technai G2 F20 STWIN, Hillsboro, OR, USA) analysis. Figure 4a–c and 4e–g shows the HR-TEM images of the ZCO-6h and ZCO-12h samples, respectively; the attained outcomes are consistent with the FE-SEM results. The two samples are composed of numerous nanoparticles, which are the basic building blocks for the formation of respective architectures via a self-assembly process The *d*-spacing values of ZCO-6h, and ZCO-12h was calculated to be 0.466 nm (Figure 4c), and 0.467 nm (Figure 4g), respectively, which were related to the (111) direction. Furthermore, the SAED patterns of ZCO-6h and ZCO-12h are shown in Figure 4d,h, respectively. The samples show well-defined diffraction rings of a polycrystalline nature, which is reliable with the XRD analysis outcomes.

### 3.3. XPS Analysis

**Figure 5:**
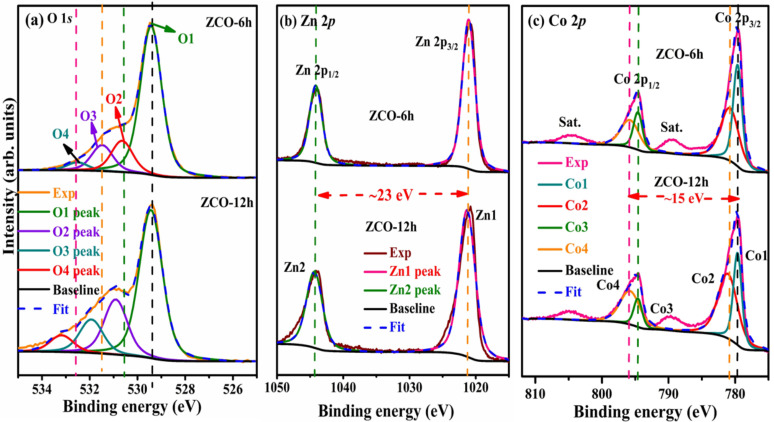
Comparison of (**a**) O *1s*, (**b**) Zn *2p* and (**c**) Co *2p* for ZCO-6h (top) and ZCO-12h (bottom).

The chemical composition of ZCO was evaluated by XPS (Thermo Scientific, K-alpha, Marietta, OH, USA). The survey of all elements in the ZCO-6h and ZCO-12h samples was studied in the order of 1350 to 0 eV, as revealed in Figure S1 (Supplementary Materials). The two samples show similar XPS profiles, which indicate their similar composition. Furthermore, the peaks correspond to the Zn, O and Co elements in the prepared materials. The presence of carbon is probably owing to the exposure of the samples to air. The O *1s* core-level spectra (Figure 5a); as depicted, the profiles of the samples are not similar. In the case of ZCO-6h, the O 1s spectrum exhibit peaks at O1—529.4 eV and O2—530.6 eV corresponding to the metal–oxygen band, whereas the O3—531.48 eV component is usually related to a low oxygen dexterity and O4—532.5 eV was assigned to the surface hydroxyl groups [38]. In ZCO-12h, the peaks appeared at O1—529.4, O2—530.9, O3—531.9 and O4—533.1 eV. The Zn *2p* doublet peaks appeared at around 1021.0/1021.3 eV and 1044.2/1044.4 eV in the spectra of ZCO-6h and ZCO-12h, respectively (Figure 5b). The former was assigned to the Zn *2p_3/2_* state, while the latter corresponds to the Zn *2p_1/2_* state, signifying the presence of Zn^2+^ in the normal state in the as-prepared ZCO samples. The binding energy variation between the spin–orbit interaction of the Zn *2p_3/2_* and Zn *2p_1/2_* states was projected as 23 eV, which is reliable with the earlier reports [39,40,41]. Similarly, the Co *2p* core-level spectra of all the samples are displayed in Figure 5c.

The asymmetric spectra exhibit three different regions of peaks, which are related to Co in the +2 and +3 states, along with a satellite peak. For comparison, we decomposed the spectra into two peaks for the +2 and +3 states and ignored the satellite peaks. The two peaks originating from the spin–orbit of Co *2p_3/2_* and *2p_1/2_* states appeared at 779.6/779.6 eV (Co1) and 794.6/794.6 eV (Co3) for the Co^3+^ state of ZCO-6h and ZCO-12h, respectively, while the other peaks appeared at 780.8/781.1 eV (Co2) and 795.8/795.8 eV (Co4) for Co^3+^. For all the samples and the two states (+2 and +3 of Co), the energy alteration between *2p_3/2_* and *2p_1/2_* was nearly 15 eV, which agrees well with former reports [12,25,42]. Therefore, Co was present in all the ZCO samples in two different states: +2 and +3. The detailed peak positions and corresponding atomic percentages, full width at half maxima (FWHMs) and Zn/Co ratios of the two samples are presented in Table 1.

### 3.4. BET Analysis

**Figure 6:**
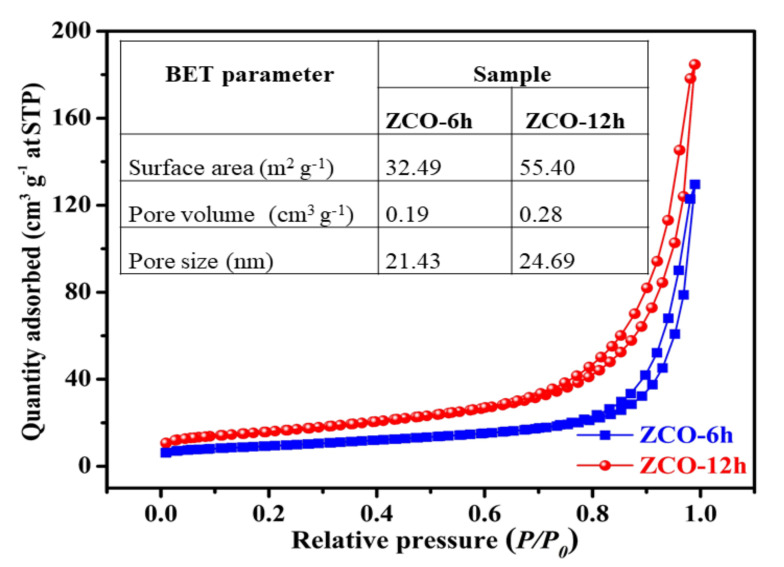
Comparison of N_2_ adsorption–desorption isotherms of ZCO-6h and ZCO-12h. The inset of the table displays a comparison of ZCO samples with respect to Brunauer Emmett Teller (BET) parameters.

The surface properties of the ZCO structures were further evaluated by Brunauer Emmett Teller (BET) investigations. N_2_ adsorption–desorption isotherms of ZCO-6h and ZCO-12h and results are presented in Figure 6. Both ZCO-6h and ZCO-12h samples exhibited typical type-IV isotherm characteristics with a type H3 hysteresis loop (Figure 6), which indicates that the samples have a characteristic mesoporous structure [16,19]. Fascinatingly, the ZCO-12h sample exhibits the highest surface area of 55.40 m^2^·g^−1^, which is much bigger than that of the ZCO-6h sample (32.49 m^2^·g^−1^). ZCO-12h possesses more places and networks, thereby generating a greater interaction area among the electrolyte and the active material owing to its large active surface area. In addition, the pore volumes of the ZCO-6h and ZCO-12h samples are 0.19 and 0.28 cm^3^·g^−1^, respectively. The larger pore volume of ZCO-12h causes less resistance to the process of diffusion of electrolyte into the active material and mitigates volume expansion during the galvanostatic charge–discharge (GCD) method [17,43]. The average pore sizes of the ZCO-6h and ZCO-12h samples were calculated as 21.43 and 24.69 nm, respectively. For the two samples, the pore size is in the 2–50 nm range, suggesting that the two samples are mesoporous nature. Typically, the size of the pores about 24.69 nm is mainly produced by PVP through the synthesis and annealing process, which is also well agreement with the FE-SEM outcomes. Based on these results, ZCO-12h is expected to show good electrochemical performances.

### 3.5. Electrochemical Analysis

**Figure 7:**
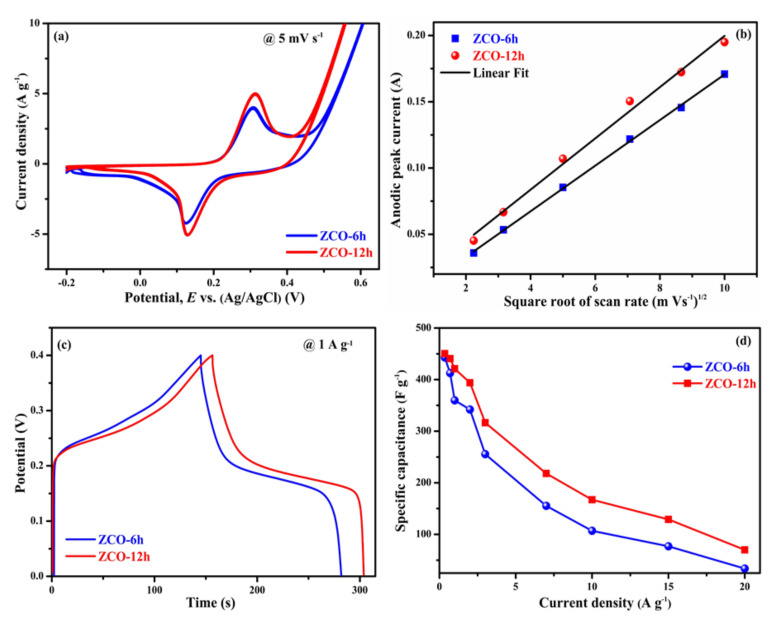
Comparison of electrochemical properties of ZCO-6h and ZCO-12h (**a**) cyclic voltammetry (CV) curves (**b**) Square root of scan rate vs. anodic peak current, (**c**) charge/discharge curves and (**d**) specific capacitance vs. current density.

Figure 7a displays the CV profiles of the two electrodes measured at a scan rate of 5 mV·s^−1^ in the potential window of −0.2 to 0.6 V (vs. Ag/AgCl) using 6 M of KOH. For the two samples, a couple of redox peaks can be detected in the CV profiles, which implies the pseudocapacitive behavior of the two electrodes rather than an EDLC (rectangular shape) [44]. The integral part surrounded through the CV curve and the current responses of the ZCO-6h and ZCO-12h electrodes almost exhibit similar trends. To further understand the processes involved in electrochemical reactions of ZCO-6h and ZCO-12h electrodes, we investigated the dependence between peak current density (*i*) and scan rate (*v*) using a power law [45].

*i* = *av*^*b*^ (1)

where ‘*i*’ is the peak current and ‘*v*’ is the scan rate, respectively, ‘*a*’ and ‘*b*’ are the appropriate constants. The value of the constant determines the charge storage process involved in the reaction. When b = 0.5, designates diffusion-controlled electrochemical process and when b = 1, denotes a non-diffusion-controlled surface redox process [45]. Figure 7b, ZCO-6h and ZCO-12h electrodes have anodic peaks with b values of 0.55 and 0.45, respectively. These values are close to 0.5, which confirms that the electrochemical process of ZCO-6h and ZCO-12h electrodes are mainly the diffusion-controlled processes ( Supplementary Materials Figure S2). In addition, the diffusion-controlled process plot clearly shows a higher current response of the ZCO-12h electrode compared to ZCO-6h, suggesting that ZCO-12h has an excellent electrochemical performance. Figure 7c shows the assessment of GCD curves for the ZCO electrodes at 1 A·g^−1^ in the potential window of 0–0.4 V. As observed from the CD measurements of the two electrodes, the curves are symmetric in shape (voltage plateaus), which indicates an excellent pseudocapacitive behavior ( Supplementary materials Figure S3). Clearly, ZCO-12h exhibits a slightly higher time response than that of the ZCO-6h electrode.

Specific capacitance (*C*s in F·g^−1^) values for ZCO-6h and ZCO-12h were calculated based on the following equation for a nonlinear charge/discharge curve [39]:

(2) $$C_{S}=\frac{2I\int Vdt}{m{\left(V_{f}-V_{i}\right)}^{2}}$$

where *I* is the current of discharge curve in mA, ∫*V*d*t* is the area under the discharge curve in V.s, *V*_f_ − *V*_i_ is the potential difference between final and initial voltage in volts (v) and m is the active electrode mass in grams (g). Based on the above equation, the specific capacitances of ZCO-6h and ZCO-12h were calculated as approximately 359.85 F·g^−1^ (28.78 C·g^−1^) and 421.05 F·g^−1^ (31.52 C·g^−1^) at 1 A·g^−1^, respectively. The determined specific capacitance values for corresponding current densities are presented in Figure 7d. The ZCO-12h electrode displays higher specific capacitance values compared to the ZCO-6h, as well as previously reported materials (Table 2) due to the morphology and its excellent BET parameters (described in the BET section). Typically, at higher current densities, the electronic and ionic transport rates of the two electrodes increased, leading to a decrease in the effective interface between the ion and electrodes, thus reducing the specific capacitance [46]. Table 2 shows the electrochemical performance of the earlier reported spinel ternary metal oxide-based electrodes. From the table, the specific capacitance of the flower-like ZnCo_2_O_4_ (ZCO-6h) and flake-like ZnCo_2_O_4_ (ZCO-12h) electrodes in this study is comparable to those of the spinel ternary metal oxide-based electrodes with a range of morphologies stated elsewhere.

**Figure 8:**
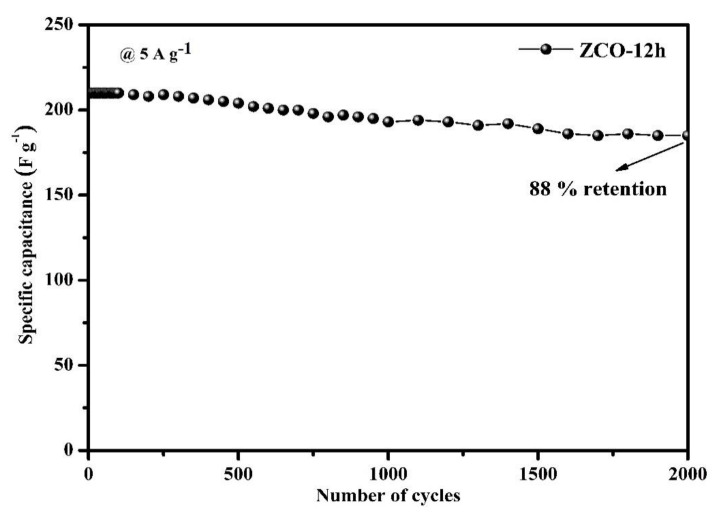
Cyclic performance of the ZCO-12h electrode.

Figure 8 displays the cyclic performance of ZCO-12h at a constant current density of 5 A·g^−1^ for 2000 cycles. Surprisingly, 88% of the maximum capacity is retained, which demonstrates the excellent cycle performance of the ZCO-12h.

**Figure 9:**
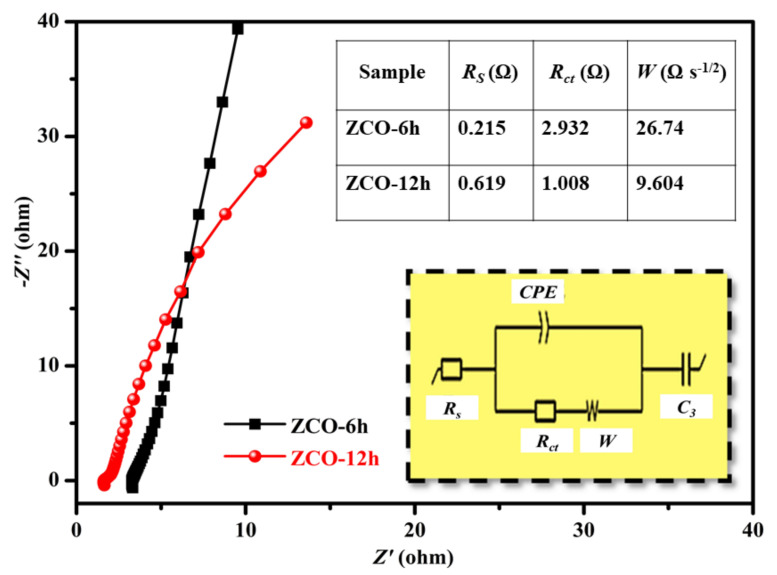
Nyquist plot for ZCO-6h and ZCO-12h. These profiles are fitted to an equivalent electronic circuit (inset) and the obtained parameter values are summarized in the inset table.

Furthermore, the electrochemical performances of the ZCO electrodes were determined through EIS analysis. The Nyquist plots (Figure 9) of the ZCO-6h and ZCO-12h electrodes were fitted to an equivalent circuit (inset of Figure 9). Typically, the impedance spectra show a semicircle in the high-frequency section, which denotes the charge–transfer resistance (*R*_ct_) produced by the Faradaic reactions and a straight line in the low-frequency section, which shows the Warburg resistance (W) related with the flow of electrolyte into the electrode surface [17,34,47]. The fitted *R*_s_, *R*_ct_ and W values of ZCO-6h and ZCO-12h are presented in the inset table of Figure 9. Noticeably, the *R*_ct_ decreases as a function of reaction temperature and the *R*_ct_ of ZCO-12h (1.008 Ω) is lower than that of ZCO-6h (2.932 Ω); this indicates that ZCO-12h has excellent ionic conductivity and a fast charge–transfer rate [48,49]. Besides, ZCO-12h exhibits a fast ion transmission in the electrolyte and a capacitive performance because of the lower diffusion resistance indicated by the ideal straight line at the low-frequency region (lower than ZCO-6h).

## 4. Conclusions

The 3D-hierarchical peony-like ZnCo_2_O_4_ structures with 2D-nanoflakes were effectively synthesized via the hydrothermal method. SEM analysis revealed that the prepared structures consist of a rough, porous surface, assembled with numerous nanoflakes and composed of nanolamella, which affords the highest specific capacitance of 421.05 F·g^−1^ (31.52 C·g^−1^) 1 A g^−1^ (for ZCO-12h). Furthermore, the maximum capacitance (88% retention for ZCO-12h) was attained after 2000 continuous charge–discharge cycles, signifies the better-cycled life of the prepared electrode martial.

## Figures and Tables

**Table 1:** Atomic percentages of fitted high-resolution O *1s*, Zn *2p* and Co *2p* spectra for ZCO-6h and ZCO-12h.

Sample	Peak Binding Energy (eV ± 0.1)/ (Relative Atomic Concentration (%))/FWHM (eV)										Ratio	Elemental at%		
	O *1s*				Zn *2p*		Co *2p*				Zn/Co	O	Zn	Co
							Co^3+^		Co^2+^					
	O1	O2	O3	O4	Zn1	Zn2	Co1	Co3	Co2	Co4				
**ZCO-6h**	529.4 (41.24) [1.05]	530.6 (8.66) [1.05]	531.5 (7.20) [1.05]	532.5 (2.23) [1.05]	1021.0 (20.17) [2.26]	1044.2	779.6 (7.87) [1.60]	794.6	780.8 (12.63) [3.26]	795.8	0.984	59.33	20.17	20.5
**ZCO-12h**	529.4 (34.10) [1.15]	530.9 (12.67) [1.15]	531.9 (7.71) [1.15]	533.1 (3.84) [1.15]	1021.3 (21.56) [2.89]	1044.4	779.6 (5.78) [1.50]	794.6	781.1 (14.34) [3.50]	795.8	1.071	58.32	21.56	20.12

**Table 2:** Comparison of supercapacitors materials based on spinel ternary metal oxides.

Material	Synthesis Method	Morphology	Specific Capacitance (F·g^−1^ @ 1 A·g^−1^)	[Ref.]
NiCo_2_O_4_	Hydrothermal	Microsphere	327	[19]
CuCo_2_O_4_	Solution combustion	Cauliflower	338	[20]
MnCo_2_O_4_	Hydrothermal	Nanowire	349.8	[22]
MgCo_2_O_4_	Molten salt method	particle	321	[23]
CoMn_2_O_4_	Solvothermal	Flower-like	321	[24]
MnCo_2_O_4_	Solvothermal	Nanosheet	346	[35]
ZnCo_2_O_4_	Hydrothermal	Nanosheet	290.5	[39]
ZnCo_2_O_4_(ZCO-6h)	Hydrothermal	Flower-like	359.85	Present work
ZnCo_2_O_4_(ZCO-12h)	Hydrothermal	Flake-like	421.05	Present work

## Supplementary Materials

The following are available online at https://www.mdpi.com/2079-4991/10/6/1206/s1, Figure S1: XPS survey spectra for (a) ZCO-6 h and (b) ZCO-12 h, Figure S2: CV curves at various scan rates for ZCO-6 h (a), ZCO-12 h (b), and Figure S3: CD curves at various current densities for ZCO-6 h (a), ZCO-12 h (b).
