# Supplementary material for: Enhanced Supercapacitive Performance of Higher-Ordered 3D-Hierarchical Structures of Hydrothermally Obtained ZnCo2O4 for Energy Storage Devices
Source: Nanomaterials (Basel). 2020 Jun 19;10(6):1206. doi: 10.3390/nano10061206 (PMC7353117; doi:10.3390/nano10061206)
Supplement: Supplementary file 1 [file nanomaterials-10-01206-s001.pdf]

Supplementary Materials

Enhanced Supercapacitive Performance of Higher-Ordered 3D Hierarchical Structures of Hydrothermally Obtained $ZnCo_2O_4$ for Energy Storage Devices

G. Rajasekhara Reddy [1], Nadavala Siva Kumar [2,*], B. Deva Prasad Raju [3,*], Gnanendra Shanmugam [4,*], Ebrahim H. Al-Ghurabi [2] and Mohammad Asif [2]

[1] Department of Instrumentation, Sri Venkateswara University, Tirupati 517502, India; gutturajasekharareddy@gmail.com
[2] Department of Chemical Engineering, King Saud University, P.O. Box 800, Riyadh 11421, Saudi Arabia; alghurabi83@windowslive.com (E.H.A.-G.); masif@ksu.edu.sa (M.A.)
[3] Department of Physics, Sri Venkateswara University, Tirupati 517 502, India
[4] Department of Biotechnology, Yeungnam University, Gyeongsan, Gyeongbuk 38541, Korea
* Correspondence: snadavala@ksu.edu.sa (N.S.K.); drdevaprasadraju@gmail.com (B.D.P.R.); gnani.science@gmail.com (G.S.)

Received: 2 May 2020; Accepted: 15 June 2020; Published: 19 June 2020

1. Materials

All chemicals were analytical grade and used directly after purchase. Nickel (Ni) foam was acquired from MTI Corporation, USA. Zinc nitrate hexahydrate [$Zn(NO_3)_2.6H_2O$], cobalt nitrate hexahydrate [$Co(NO_3)_2.6H_2O$], polyvinylpyrrolidone [PVP (K-30), $(C_6H_9NO)_n$)], urea [$CO(NH_2)_2$], polyvinylidene difluoride [PVDF, -$(C_2H_2F_2)_n$-], N-methyl-2-pyrrolidone (NMP, C_5H_9NO), carbon black, and potassium hydroxide (KOH) were procured from Sigma-Aldrich, USA. Deionized (DI) water with a resistivity of 18.2 MΩ.cm was used as a solvent for all experiments。

2. Material Characterization

The crystallinity of the prepared powders was studied using an X-ray diffractometer (PANalytical X'Pert PRO) with Cu K_α radiation (1.54 Å) operated at 40 kV and 30 mA. The surface morphology and elemental composition of the samples were determined using a field emission scanning electron microscope coupled with an energy-dispersive X-ray spectroscope (FE-SEM, S-4800, Hitachi, Japan). The nanostructures of the powders were examined by high-resolution transmission electron microscopy (HRTEM, Technai G2 F20 STWIN, USA) performed at 200 kV. The chemical compositions of the prepared samples were investigated by X-ray photoelectron spectroscopy (XPS, K-alpha, Thermo Scientific, USA). The samples were excited using a monochromatic Al K_α X-ray radiation (1486.6 eV), and the data were recorded and processed using commercial software A vantage (version 5.932, Thermo Scientific, USA). Furthermore, N_2 absorption-desorption curves were obtained using a surface analyzer (3-Flex, Micromeritics, USA).

3. Electrode Preparation and Electrochemical Measurements

For the three-electrode electrochemical station, the working electrode was fabricated as follows: the active materials (ZCO-6h/ZCO-12h) were well dispersed in NMP along with PVDF and carbon black at a weight ratio 80:10:10, respectively. The mixture was ultrasonicated for 30 min to drop-cast on a Ni foam. For this, 2.5 × 1 cm^2 Ni foams were first ultrasonically cleaned with a concentrated HCl solution to eliminate the surface layer, and then with deionized water and pure ethanol for 10 min each. Then, they were dried in a vacuum oven at 80 °C for 6 h. Finally, approximately 8 mg of the prepared mixture was coated onto the Ni foam over a working area of 1.5 × 1 cm^2 and dried at 80 °C

for 6 h. Electrochemical measurements were carried out on an electrochemical workstation (CHI 760 E, CH instruments, USA) at room temperature (RT). For all the experiments, Ag/AgCl, platinum wire and as-prepared Ni electrode were used as the reference, counter and working electrodes, respectively, while KOH was used as the active electrolyte. Cyclic voltammetry (CV), galvanostatic charge–discharge cycling (GCD) and electrochemical impedance spectroscopy (EIS) were performed to examine the electrochemical performances of the samples.

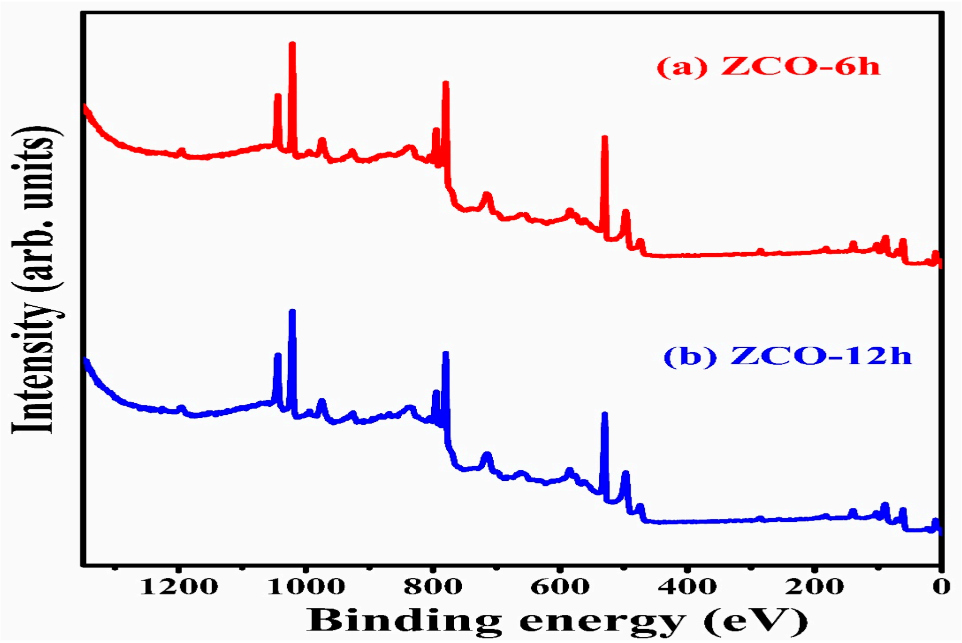

Figure S1. XPS survey spectra for (a) ZCO-6 h and (b) ZCO-12 h.

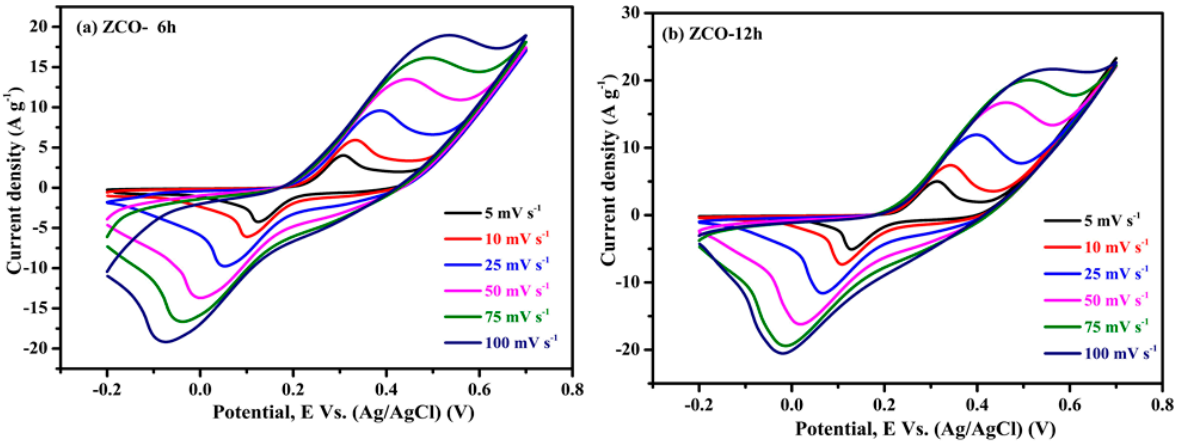

Figure S2. CV curves at various scan rates for ZCO-6 h (**a**), ZCO-12 h (**b**).

4. Abbreviations

XRD: X-ray diffraction; FE-SEM: Field emission scanning electron microscopy; EDS: Energy-dispersive X-ray spectroscopy; HRTEM: High-resolution transmission electron microscopy; SAED: Selected area electron diffraction; XPS: X-ray photoelectron spectroscopy; BET: Brunauer Emmett Teller; CV: Cyclic Voltammetry; GCD: Galvanostatic charge-discharge; EIS: Electrochemical Impedance Spectroscopy.

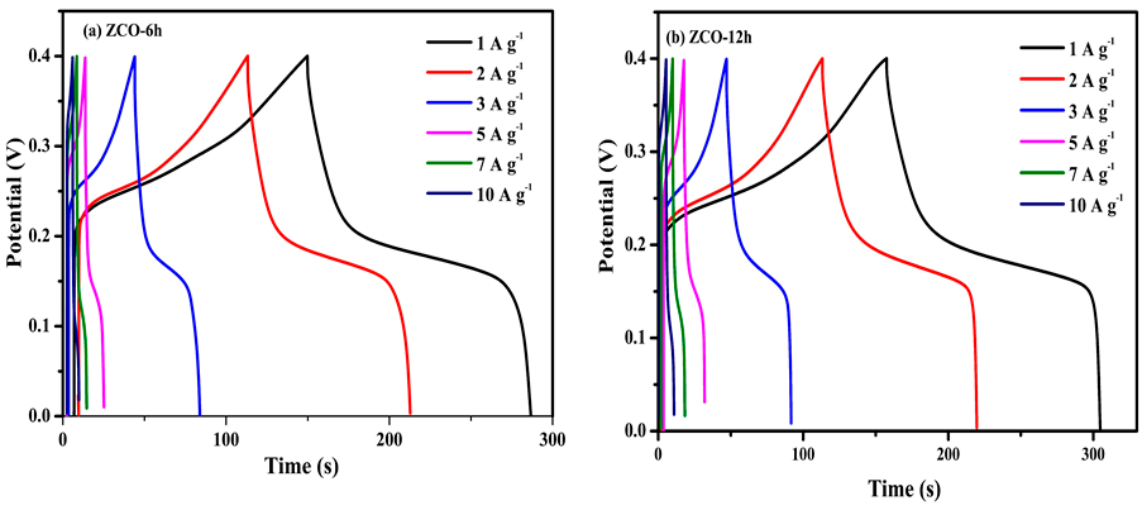

Figure S3. Charge-discharge (CD) curves at various current densities for ZCO-6 h (**a**), ZCO-12 h (**b**).
